# Volatile 1-octanol of tea (*Camellia sinensis* L.) fuels cell division and indole-3-acetic acid production in phylloplane isolate *Pseudomonas* sp. NEEL19

**DOI:** 10.1038/s41598-021-82442-7

**Published:** 2021-02-02

**Authors:** Poovarasan Neelakandan, Chiu-Chung Young, Asif Hameed, Yu-Ning Wang, Kui-Nuo Chen, Fo-Ting Shen

**Affiliations:** 1 Department of Soil & Environmental Sciences, College of Agriculture and Natural Resources, National Chung Hsing University, Taichung, 40227 Taiwan, ROC; 2 Innovation and Development Center of Sustainable Agriculture (IDCSA), National Chung Hsing University, Taichung, 40227 Taiwan, ROC; 3 Yenepoya Research Centre, Yenepoya Deemed to be University, Mangalore, 575018 India

**Keywords:** Biochemistry, Biotechnology, Microbiology, Plant sciences, Ecology

## Abstract

Tea leaves possess numerous volatile organic compounds (VOC) that contribute to tea’s characteristic aroma. Some components of tea VOC were known to exhibit antimicrobial activity; however, their impact on bacteria remains elusive. Here, we showed that the VOC of fresh aqueous tea leaf extract, recovered through hydrodistillation, promoted cell division and tryptophan-dependent indole-3-acetic acid (IAA) production in *Pseudomonas* sp. NEEL19, a solvent-tolerant isolate of the tea phylloplane. 1-octanol was identified as one of the responsible volatiles stimulating cell division, metabolic change, swimming motility, putative pili/nanowire formation and IAA production, through gas chromatography-mass spectrometry, microscopy and partition petri dish culture analyses. The bacterial metabolic responses including IAA production increased under 1-octanol vapor in a dose-dependent manner, whereas direct-contact in liquid culture failed to elicit such response. Thus, volatile 1-octanol emitting from tea leaves is a potential modulator of cell division, colonization and phytohormone production in NEEL19, possibly influencing the tea aroma.

## Introduction

Tea is one of the most popular beverages in the world, ranked second among non-alcoholic drinks. The non-volatile and volatile compounds respectively determine the taste and aroma of tea^1^. Extensive research has been done characterizing volatile and non-volatile compounds, which together govern the flavor and quality of tea. Organic acids, sugars, free amino acids have been commonly identified in the non-volatile pool, whereas aldehydes, alcohols, ketones, sesquiterpenes and furans were found in the volatile fraction of tea^1–9^.

Volatile organic compounds (VOC) released by plants can shape plant-associated microbial communities by exerting growth-inhibiting and/or promoting attributes^10,11^. Plant VOC usually exhibit antimicrobial properties and thought to act as defense agents^10,12–16^. Plant VOC can repel herbivores or attract herbivore predators^17^. VOC such as methanol and monoterpenes were known to serve as the carbon source for plant-associated bacteria^10,18–20^.

The interaction between plant VOC and phyllosphere microbes are bidirectional, and hence deserves more attention^10^. VOC composition of plants can be manipulated by plant-associated microbes and herbivorous insects^11,21^. To date, around 600 volatile compounds have been reported from tea^1^. VOC of tea phyllosphere exhibited insect attractant^22,23^ and antimicrobial attributes^24^. However, the impact of tea VOC on bacteria that colonize the phylloplane remains unknown.

Some strains of *Pseudomonas* tolerate organic solvents in liquid cultures^25–29^. Representatives of *Pseudomonas* are one of the most abundant taxa colonizing the plant leaves^30^. Indole-3-acetic acid (IAA), a phytohormone capable of controlling several aspects of plant growth and development, can be synthesized by some *Pseudomonas*^31,32^. IAA modulates secondary metabolism and adventitious root formation in plants^33^. It mediates plant–microbe relationship and influences bacterial physiology^32,34^. A better understanding of chemical crosstalk between plant VOC and bacterial IAA producer may allow enhancement of tea plant growth and physiology, leading to improved flavor.

Here, we hypothesized that the solvent-tolerant strain associated with tea leaves can sense VOC and self-modulate the biofilm and phytohormone formation. The hypothesis was tested by using an alcohol-tolerant phylloplane isolate *Pseudomonas* sp. NEEL19 and crude aqueous tea extract (TeaAq) that emit volatiles.

## Results

### Molecular identification and phylogenetic characterization

NEEL19 shared 100% 16S rRNA gene sequence similarity with *Pseudomonas juntendi* BML3^T^, a bacterium of clinical origin^35^. It also shared ≥ 99.0% sequence similarities with sixteen *Pseudomonas* species and several genome-sequenced isolates (Table S1). However, in the neighbor-joining tree, NEEL19 established a distinct and strong phyletic lineage with *P. alloputida* VKh7^T^ (99.8% similarity; 74% bootstrap support), isolated from the bean rhizosphere^36^ (Figure S1). Phylogenetic linkages were also seen establishing with a clinical isolate *P. mosselii* CIP 105259^T^^37^, and solvent-tolerant *P. putida* strains BIRD-1^25^, S12^27,38^ and KT2440^39^. Thus, NEEL19 was identified as a Pseudomonad, but a species-level distinction was not possible due to the limited variation in inter-species and intra-species 16S rRNA gene sequences in this diverse genus.

### Biochemical and enzymatic characterization

Experiments were performed to identify plant growth-promotive traits and nutritional requirements of NEEL19. Cells reduced nitrate, produced IAA and siderophore (Figure S2a), but lacked diazotrophic and DNase (Figure S2b) activities. Cells showed catalase, cytochrome oxidase, amylase, arginine dihydrolase, alkaline phosphatase, esterase (C 4), esterase lipase (C 8), lipase (C 14), leucine arylamidase, acid phosphatase, naphthol-AS-BI-phosphohydrolase and *β*-glucosidase activities; assimilated D-glucose, L-arabinose, D-mannose, potassium gluconate, capric acid, malic acid, trisodium citrate, D-melibiose and L-arabinose; oxidized most (74/95) of carbon sources present on GN2 microplate (Supplementary Information). Cells were sensitive exclusively to levofloxacin (2–4 mg L^−1^) and high doses of gentamicin and norfloxacin (8 mg L^−1^) among tested antibiotics (Table S2). This phenotypic analysis suggests that NEEL19 is a Pseudomonad with some plant growth-promoting features typical of many isolates from the phytosphere.

### Impact of alcohols on cell growth and morphology of NEEL19

**Figure 1:**
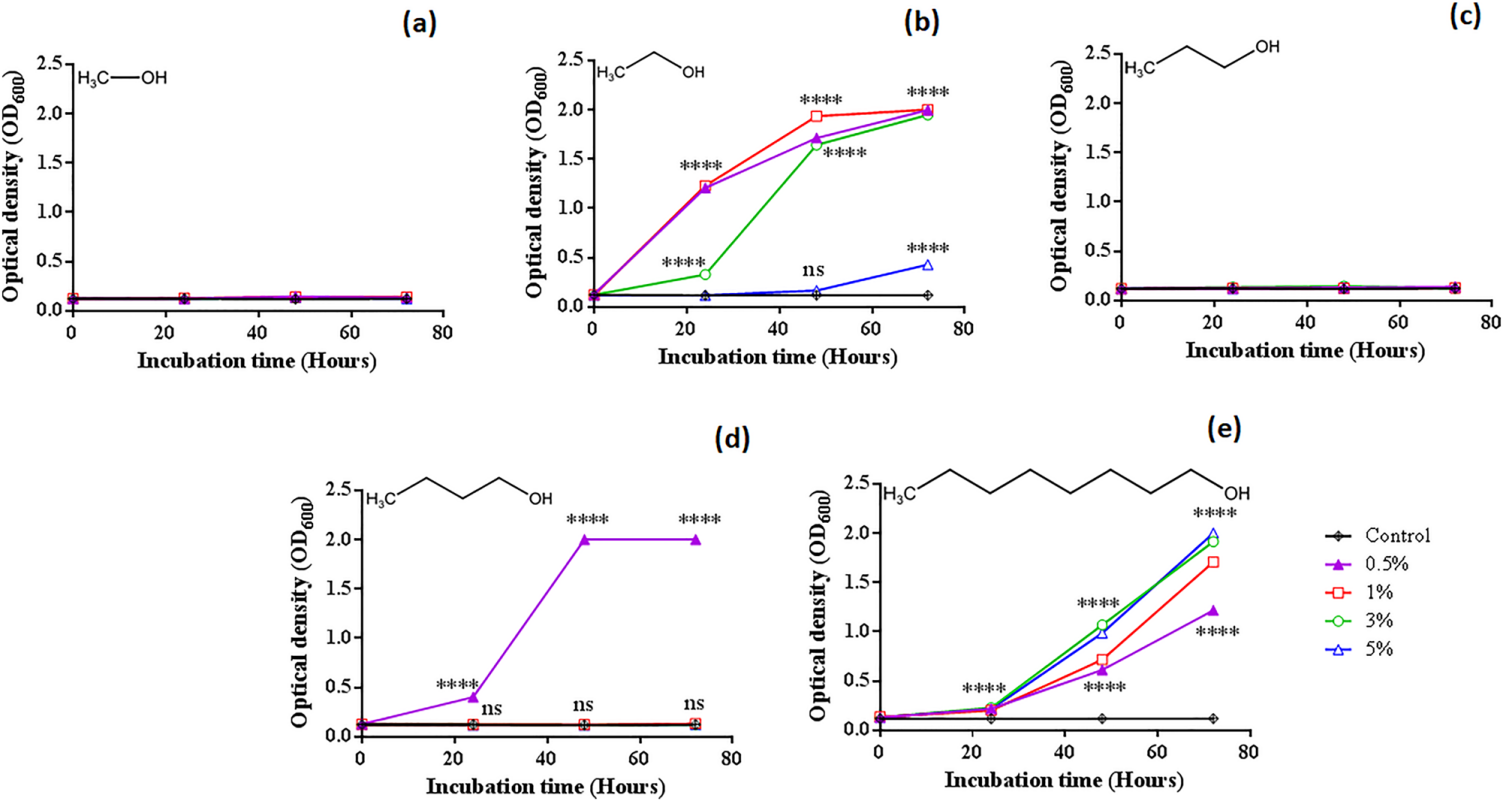
Growth kinetics of *Pseudomonas* sp. NEEL19 under the direct contact of 0.5‒5% (v/v) alcohols when supplied as sole energy and carbon sources in DSMZ 125*. Growth pattern in (**a**) methanol, (**b**) ethanol, (**c**) 1-propanol, (**d**) 1-butanol and **(e**) 1-octanol are shown. Inoculated DSMZ 125* without respective carbon source was used as a control. OD_600_, optical density at 600 nm. Error bar, mean (n = 3) ± s.d., are smaller than symbols. ****P < 0.0001; ns, non-significant. Dose: Open-diamond, control (0%); filled-triangle, 0.5%; open-square, 1%; open-circle, 3%; open-triangle, 5%.

Alcohol-tolerance in NEEL19 was assessed since this isolate shared phylogenetic proximity with several solvent tolerant strains. Cell growth was tested by supplementing liquid basal medium (DSMZ 125*, see methods) with 0, 0.5, 1, 3 and 5% (v/v) methanol, ethanol, 1-propanol, 1-butanol and 1-octanol as sole carbon and energy sources. Growth was not detected at 0–5% methanol (Fig. 1a), whereas significant (*P* < 0.0001) growth occurred at 0.5–3% ethanol within 24 h, yielding distinct trendlines (Fig. 1 b). However, 5% ethanol was repressive as visible growth occurred only at 72 h. Cells failed to grow at 0–5% 1-propanol (Fig. 1c), whereas significant (*P* < 0.0001) growth occurred at 0.5% 1-butanol within 24 h, yielding a typical sigmoidal trendline (Fig. 1d). Higher (> 0.5%) doses of 1-butanol failed to stimulate growth. In contrast, significant (*P* < 0.0001) growth occurred at 0.5–5% 1-octanol at 24 h with subsequent dose-dependent exponential increments (Fig. 1e). Thus, alcohols were identified to differentially influence the cell growth of NEEL19.

Impacts of various alcohols (0.5%, v/v) on cell morphology of NEEL19 were investigated. Cells appeared elongated with smooth surfaces in methanol and ethanol (Figure S3a and b); smooth and stunted in 1-propanol (Figure S3c); irregular and stunted in 1-butanol and 1-octanol (Figure S3d and e). Mean cell length reduced significantly (*P* < 0.0001) in 1-propanol (1.31 ± 0.12 µm), 1-butanol (1.32 ± 0.10 µm) and 1-octanol (1.30 ± 0.11 µm) as compared to ethanol (1.67 ± 0.08 µm) (Figure S4a). Similarly, mean cell width reduced significantly in 1-propanol (0.65 ± 0.08 µm, *P* < 0.01), 1-butanol (0.68 ± 0.08 µm, *P* < 0.01) and 1-octanol (0.73 ± 0.02 µm, *P* < 0.1) as compared to ethanol (0.84 ± 0.07 µm) (Figure S4b). Taken together, various alcohols as growth substrates in liquid cultures found to alter OD, shape, size and surface features of bacterial cells.

### Chemical characterization of crude aqueous extract of fresh tea leaves (TeaAq)

**Figure 2:**
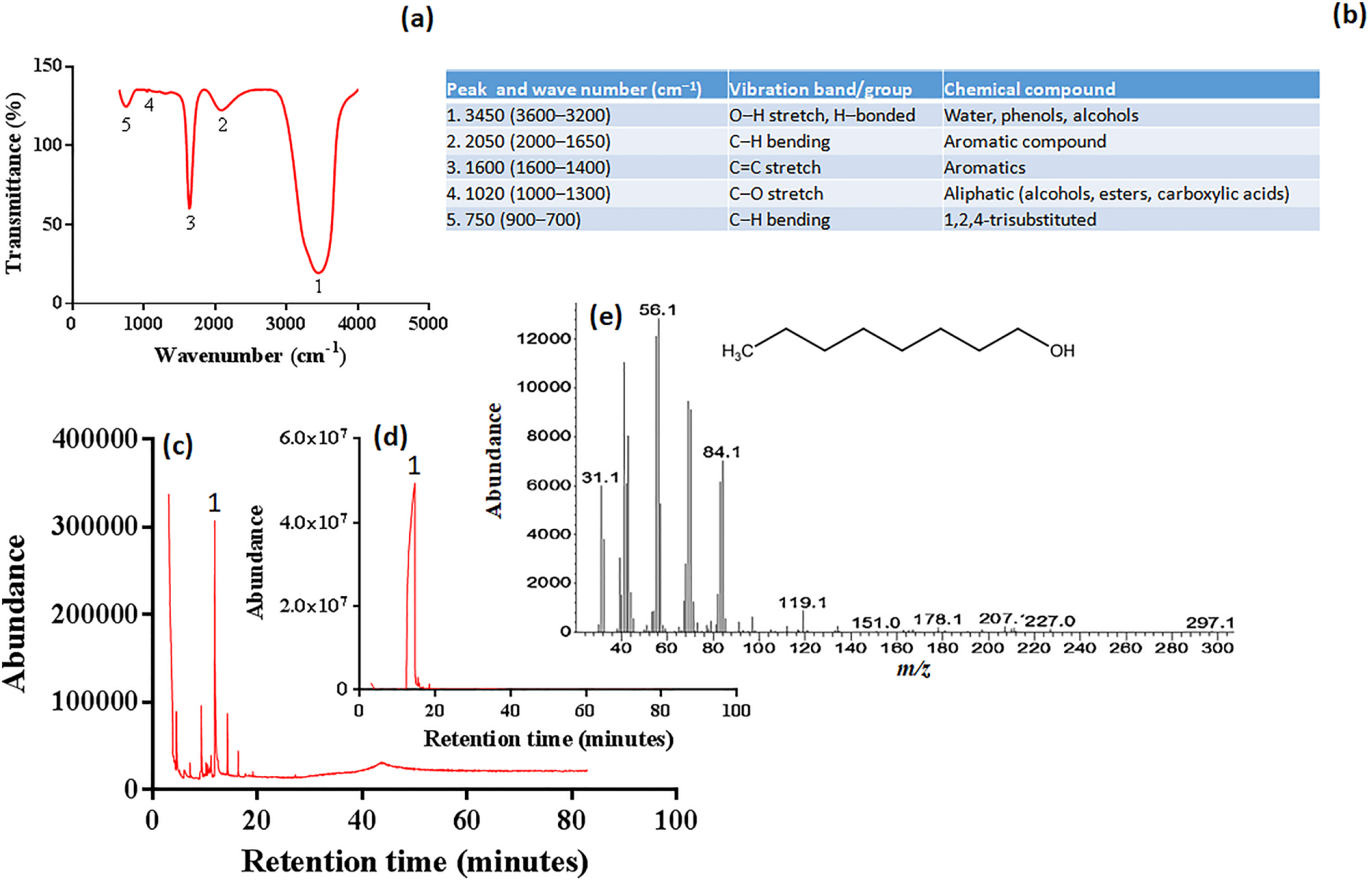
FT-IR and GC–MS analyses of aqueous tea extract (TeaAq). (**a**) FT-IR spectrum, (**b**) IR vibrational band summary, (**c**) gas chromatogram of TeaAq, (**d**) gas chromatogram of 1-octanol standard and (**e**) mass fingerprint of 1-octanol (1).

Clevenger apparatus (Figure S5) was used to extract TeaAq through hydrodistillation from tea leaves. TeaAq appeared pale yellow with a distinct odor indicating the presence of VOC. The fourier transform infrared spectrometry (FT-IR) data revealed signatory infrared vibrational bands of aromatics and alcohols in TeaAq (Fig. 2a,b). Gas chromatography-mass spectrometry (GC–MS) detected several phytochemicals including 1-octanol in TeaAq (Fig. 2c, Table S3), whereas no traces of ethanol and 1-propanol were found. The presence of 1-octanol was verified through the retention time and mass profile of standard (Fig. 2d–e).

### Impact of vapors of volatile compounds on bacteria

**Figure 3:**
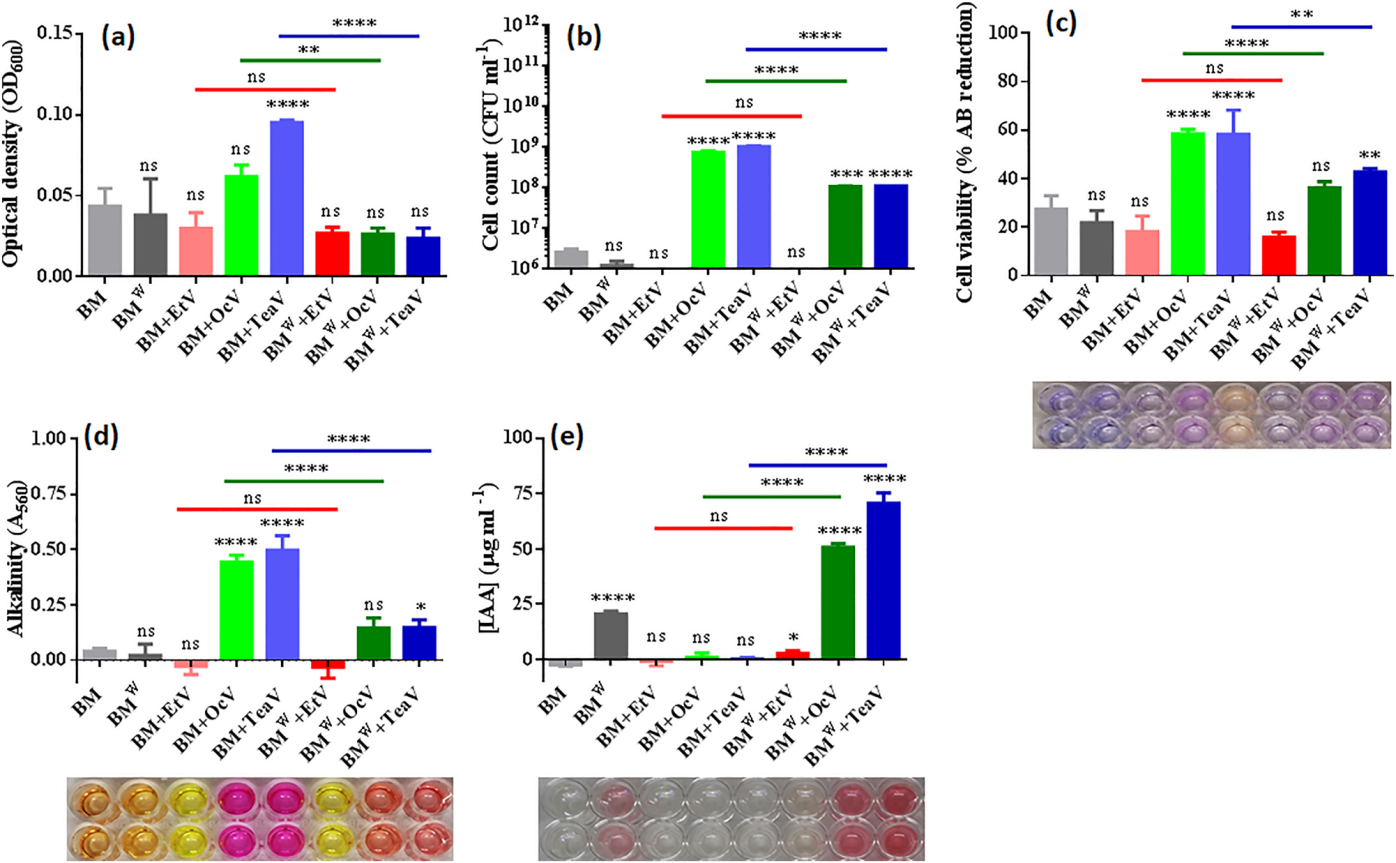
Influence of the vapors of ethanol (EtV), 1-octanol (OcV) and aqueous tea extract (TeaV) on *Pseudomonas* sp. NEEL19, cultured in DSMZ 125* without and with 0.1% (w/v) Trp (BM and BM^W^, respectively). (**a**) optical cell density (OD_600_), (**b**) cultivable cell count, (**c**) cell viability, (**d**) media alkalization and (**e**) IAA production are shown; microplate sections obtained in this study (**c‒e**) represent corresponding chromogenic reactions. Inoculated Trp-fee DSMZ 125* without vapor treatment was used as a control. Error bar, mean (n = 4) ± s.d. *P < 0.1, **P < 0.05, ***P < 0.01, ****P < 0.0001; ns, non-significant. Treatment codes are defined in the section abbreviations.

Cells were inoculated into DSMZ 125* without and with 0.1% (w/v) L-tryptophan (Trp) supplement (BM and BM^W^, respectively). BM and BM^W^ were exposed to the vapors of ethanol (EtV), 1-octanol (OcV) and aqueous tea extract (TeaV) in partition petri dishes (PPD) (Figure S6). Optical cell density (OD_600_) was found to increase significantly in BM + TeaV as compared to control BM (0.10 ± 0.002 *vs* 0.04 ± 0.01, *P* < 0.0001) (Fig. 3a). OD_600_ values of BM + OcV and BM + TeaV were higher when compared to their respective Trp-containing BM^W^ + OcV (0.06 ± 0.01 *vs* 0.03 ± 0.004, *P* < 0.05) and BM^W^ + TeaV (0.10 ± 0.002 *vs* 0.02 ± 0.006, *P* < 0.0001) treatments. The number of cultivable cells (colony-forming units, CFU ml^−1^) were high in BM + OcV (7.35 × 10^8^ ± 6.35 × 10^7^, *P* < 0.0001), BM + TeaV (1.02 × 10^9^ ± 3.46 × 10^7^, *P* < 0.0001), BM^W^ + OcV (1.05 × 10^8^ ± 5.77 × 10^6^, *P* < 0.01) and BM^W^ + TeaV (1.10 × 10^8^, *P* < 0.0001) as compared to control BM (2.55 × 10^6^ ± 5.20 × 10^5^) (Fig. 3b). Higher CFU were found in BM + OcV and BM + TeaV when compared to their respective Trp-containing BM^W^ + OcV (7.35 × 10^8^ ± 6.35 × 10^7^ *vs* 1.05 × 10^8^ ± 5.77 × 10^6^, *P* < 0.01) and BM^W^ + TeaV (1.02 × 10^9^ ± 3.46 × 10^7^ *vs* 1.10 × 10^8^, *P* < 0.0001) treatments. In contrast, cells that originated from BM + EtV and BM^W^ + EtV treatments were non-cultivable. Thus, while EtV treatment ceased the cultivability of NEEL19, OcV and TeaV exposures increased the cultivable cell count, which can be suppressed significantly by Trp.

The alamarBlue (AB) dye reduction was significantly high in BM + OcV (58.4 ± 2.00%, *P* < 0.0001), BM + TeaV (58.3 ± 10.0%, *P* < 0.0001) and BM^W^ + TeaV (42.8 ± 1.38%, *P* < 0.05) as compared to control BM (27.5 ± 5.44%) (Fig. 3c). Increased dye reduction was found in BM + OcV and BM + TeaV as compared to their respective BM^W^ + OcV (58.4 ± 2.00% *vs* 36.2 ± 2.54%, *P* < 0.0001) and BM^W^ + TeaV (58.3 ± 10.0% *vs* 42.8 ± 1.38%, *P* < 0.05) treatments. Dye reduction (non-significant *vs* control BM) was also detected in BM^W^ (21.9 ± 4.88%), BM + EtV (18.2 ± 4.88%) and BM^W^ + EtV (15.8 ± 2.15%). Taken together, cells exposed to EtV were appeared to be in the viable but not cultivable stage, whereas OcV and TeaV treatments boosted viability and cultivability, which can be suppressed significantly by Trp.

Media alkalization (A_560_) and acidification (A_415_) were probed using phenol red. Colorimetric analysis of cell-free supernatant indicated significant media alkalization in BM + OcV (A_560_ 0.44 ± 0.03, pH ~ 8.0, *P* < 0.0001), BM + TeaV (A_560_ 0.50 ± 0.07, pH ~ 8.2, *P* < 0.0001) and BM^W^ + TeaV (A_560_ 0.15 ± 0.03, pH ~ 7.6, *P* < 0.1) as compared to control BM (A_560_ 0.04 ± 0.01, pH ~ pH 6.9) (Fig. 3d). The alkalization was significantly (*P* < 0.0001) high in BM + OcV and BM + TeaV as compared to their respective BM^W^ + OcV (pH ~ 8.0 *vs* pH ~ 7.5) and BM^W^ + TeaV (pH ~ 8.2 *vs* pH ~ 7.6) counterparts. In contrast, the media turned to be acidic in BM + EtV (pH ~ 6.3) and BM^W^ + EtV (pH ~ 6.2). Therefore, cell-mediated media alkalization achieved through OcV and TeaV exposures was found to be suppressed significantly by Trp.

Significant amount of IAA was detected in BM^W^ (20.6 ± 1.27 µg ml^−1^, *P* < 0.0001), BM^W^ + EtV (2.57 ± 1.29 µg ml^−1^, *P* < 0.1), BM^W^ + OcV (50.7 ± 1.71 µg ml^−1^, *P* < 0.0001) and BM^W^ + TeaV (70.7 ± 4.68 µg ml^−1^, *P* < 0.0001) as compared to control BM (not detectable) (Fig. 3e). IAA was significantly (*P* < 0.0001) higher in BM^W^ + OcV and BM^W^ + TeaV as compared to Trp-lacking BM + OcV (50.7 ± 1.71 µg ml^−1^ *vs* 0.97 ± 1.92 µg ml^−1^) and BM + TeaV (70.7 ± 4.68 µg ml^−1^ *vs* 0.3 ± 0.52 µg ml^−1^) treatments. IAA production was not detected in BM + EtV. Thus, OcV and TeaV were found to promote IAA production in NEEL19, particularly in the presence of Trp.

### Impact of vapors on cell morphology

**Figure 4:**
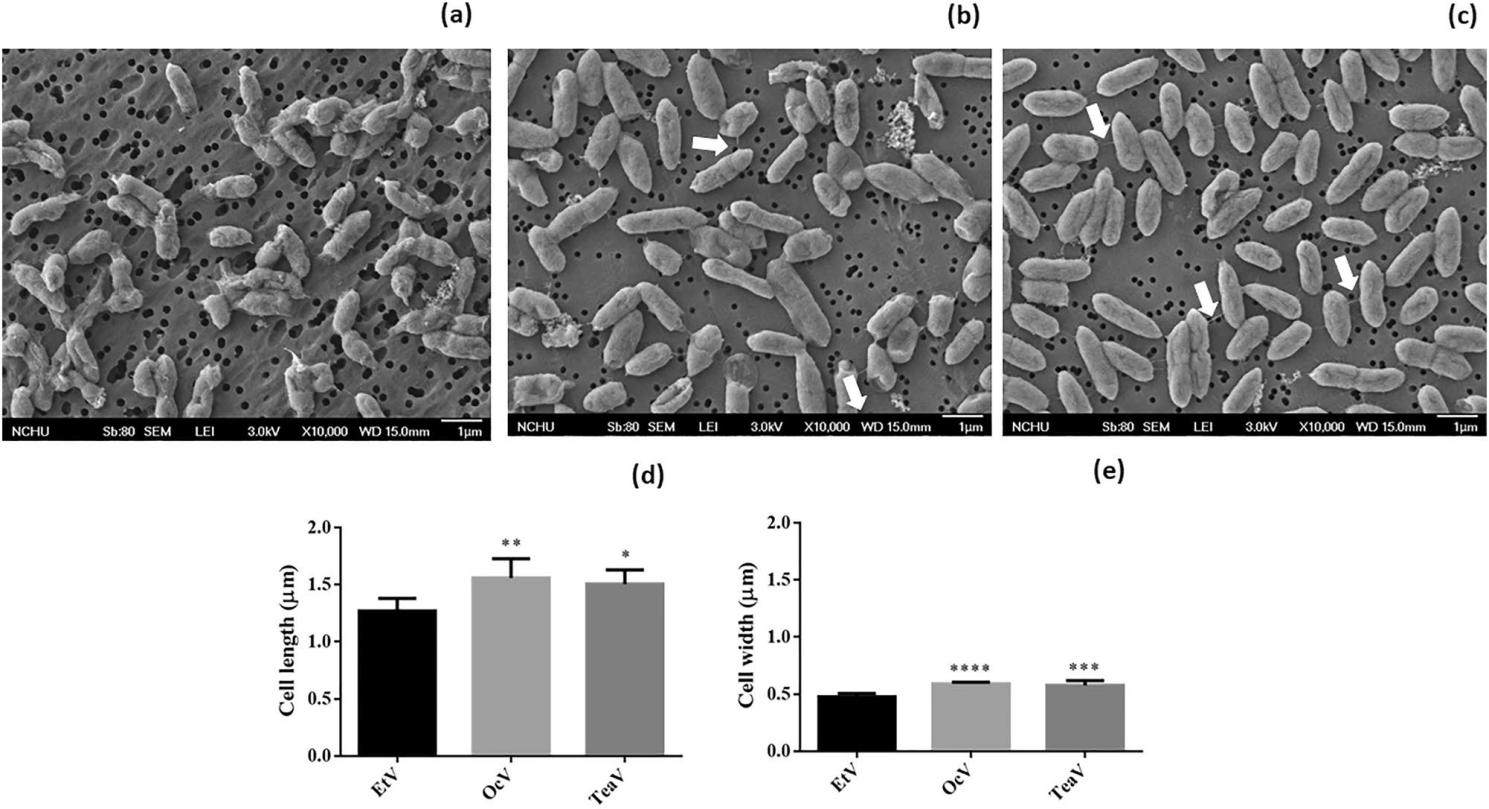
Influence of the vapors of ethanol (EtV), 1-octanol (OcV) and aqueous tea extracts (TeaV) on cell morphology of *Pseudomonas* sp. NEEL19, cultured in DSMZ 125* containing 0.1% (w/v) Trp. Scanning electron micrographs of cells originated from (**a**) EtV, (**b**) OcV and (**c**) TeaV treatments are shown. (**d**) and (**e**) show variations in cell length and width, respectively; Sample exposed to EtV was used as a control. Arrow, putative pili/nanowire; Error bar, mean (n = 60) ± s.d. *P < 0.1, **P < 0.05, ***P < 0.01, ****P < 0.0001; ns, non-significant.

EtV exposure resulted in cells with rough and irregular cell surfaces (Fig. 4a). In contrast, OcV and TeaV exposures produced cells with smooth and regular cell surface with the occasional intercellular network of putative pili/nanowires (Fig. 4b and c). Cell length increased in OcV (1.56 ± 0.17 µm, *P* < 0.05) and TeaV (1.51 ± 0.12 µm, *P* < 0.1) as compared to EtV control (1.27 ± 0.11 µm) (Fig. 4d). Similarly, cell width also increased in OcV (0.59 ± 0.02 µm, *P* < 0.0001) and TeaV (0.58 ± 0.04 µm, *P* < 0.01) when compared to EtV control (0.48 ± 0.03 µm) (Fig. 4e). Thus, OcV and TeaV exerted strikingly similar impacts on cell size and surface characteristics.

### Dose-dependent analysis of OcV

**Figure 5:**
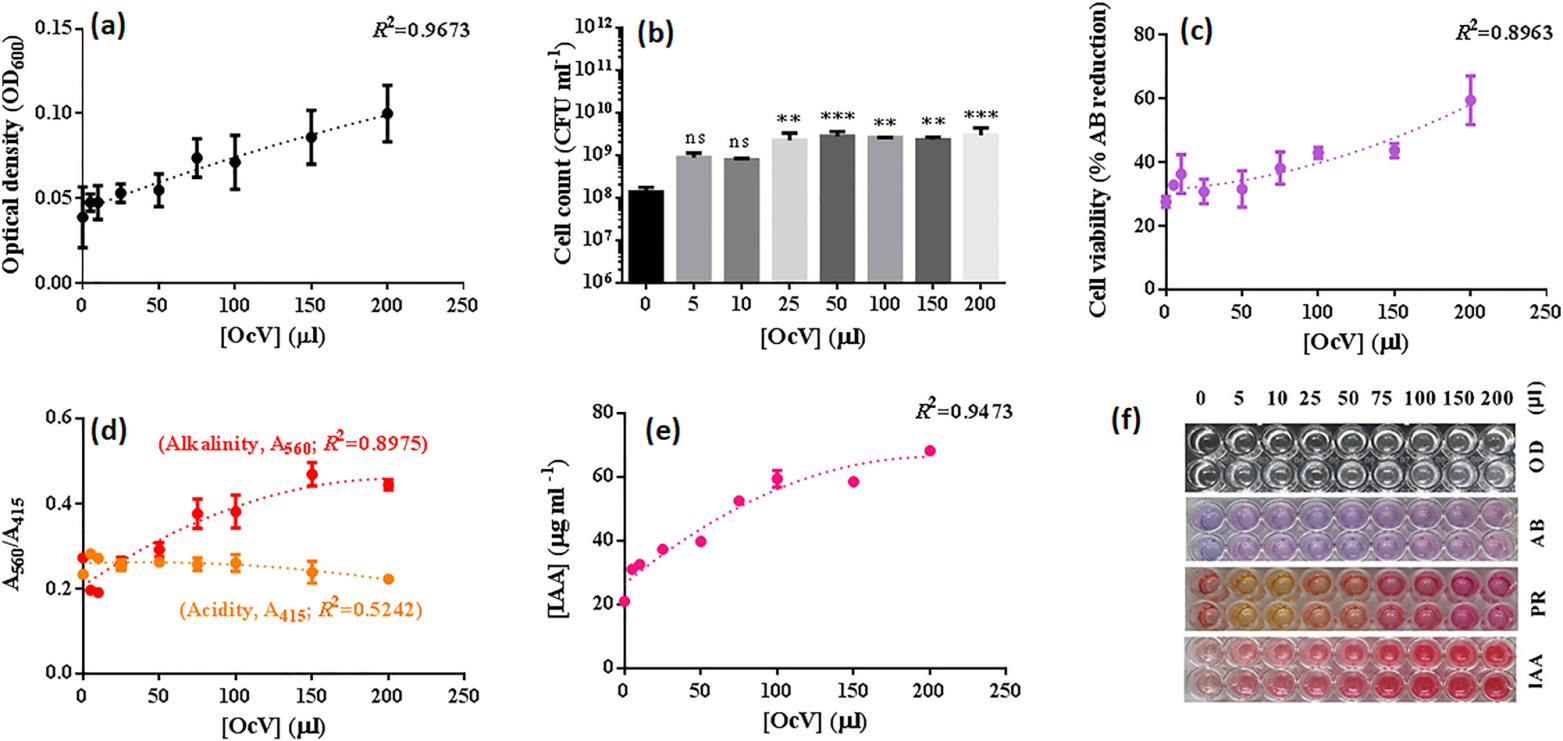
Influence of the vapors of 1-octanol (OcV) on *Pseudomonas* sp. NEEL19, cultured in DSMZ 125* containing 0.1% (w/v) Trp. Dose-dependent variation in (**a**) optical cell density (OD_600_), (**b**) cultivable cell count, (**c**) cell viability, (**d**) media acidity/alkalinity and (**e**) IAA production, when supplied with 0‒200 µl 1-octanol (plate^‒1^) as the volatile sole source of carbon and energy, are shown; BM^W^ without OcV (0 µl) exposure was used as a control in (**b**). Microplate sections obtained in this study (**f**, top-to-bottom) represent dose-dependent variation in turbidity (**a**), and chromogenic reactions of cell viability (**c**), media alkalization (**d**) and IAA production (**e**) assays. A_415_ and A_560_, absorbances at 415 and 560, respectively. IAA, indole-3-acetic acid; OD, optical cell density; PR, phenol red; AB, alamarBlue. Error bar, mean (n = 4) ± s.d. **P < 0.05, ***P < 0.01; ns, non-significant.

The impact of OcV was tested on NEEL19 in detail by using PPD cultures (Figure S6) and results are shown in Fig. 5a–f. OD increased with increasing doses (0–200 µl) of OcV (*R*^2^ = 0.9673) (Fig. 5a). Cultivable cells were significantly high at ≥ 25 µl OcV (Fig. 5b). Cell viability (*R*^2^ = 0.8963), media alkalization (*R*^2^ = 0.8975) and IAA production (*R*^2^ = 0.9473) were increased in a dose-dependent manner having distinct polynomial trendlines (Fig. 5c–e). These data strongly supported the growth-promotive attributes of volatile 1-octanol on NEEL19.

### Impact of OcV on cell motility

The colony diameter of NEEL19 reached ~ 2 cm within 36 h at 0.3% (w/v) agar, indicating rapid swimming motility on full-strength nutrient media (Figure S7a). However, swarming and twitching motilities were absent as determined at 0.5% and 1.0% agar (w/v), respectively (Figure S7b–c). In DSMZ 125* supplemented with 0.3% (w/v) agar, OcV significantly (*P* > 0.1) increased the colony diameter (1.63 ± 0.62 cm) as compared to control (1.08 ± 0.38 cm) (Figure S7d–f). Thus, volatile 1-octanol promoted the swimming motility of NEEL19.

### IAA production under direct contact of volatile compounds in liquid cultures

**Figure 6:**
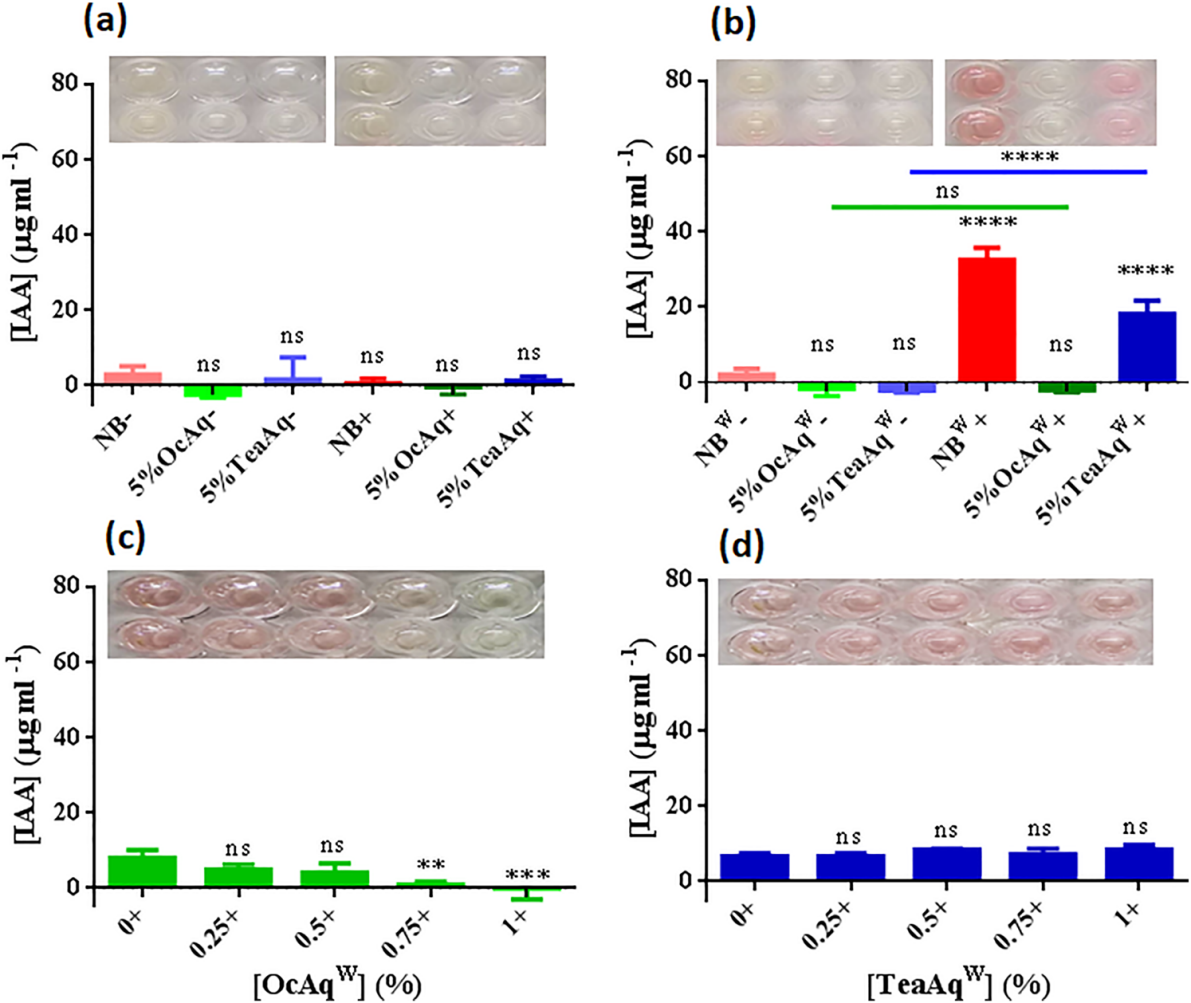
Influence of the direct contact of 1-octanol (OcAq) and aqueous tea extract (TeaAq) on IAA production in *Pseudomonas* sp. NEEL19, cultured in the presence/absence of 0.1% (w/v) Trp. (**a**) and (**b**) respectively show IAA production in the absence and presence of 0.1% (w/v) Trp when supplied with 5% (v/v) 1-octanol (OcAq) and 5% (v/v) aqueous tea extract (TeaAq). NB and NB^W^, full-strength nutrient broth (Himedia) without and with 0.1% (w/v) Trp, respectively. + , cells present; –, cells absent. Cell-free NB and NB^W^ were used as controls in (**a**) and (**b**), respectively. (**c**) and (**d**) show IAA production at low concentrations (< 1%) of OcAq and TeaAq present in DSMZ 125* with 0.1% (w/v) Trp supplement, respectively. BM^W^ without OcAq and TeaAq supplement were used as controls in (**c**) and (**d**), respectively. Microplate sections (insert, **a–d**) obtained in this study represent chromogenic reactions of respective IAA assay. Error bar, mean (n = 4) ± s.d. **P < 0.05, ***P < 0.01, ****P < 0.0001; ns, non-significant.

The formation of IAA was tested while supplying high-doses of carbon sources (full-strength NB, 5%TeaAq (v/v) and 5%OcAq (v/v)) in liquid cultures without and with Trp. No substantial IAA production occurred in the absence of Trp (Fig. 6a). In contrast, significant IAA production occurred in Trp-supplemented NB (32.6 ± 3.08 µg ml^−1^, *P* < 0.0001) and 5%TeaAq (18.2 ± 3.49 µg ml^−1^, *P* < 0.0001) as compared to control (2.15 ± 1.44 µg ml^−1^) (Fig. 6b). Trp-supplemented 5%OcAq showed no significant increase in IAA formation despite the presence of cells. In contrast, Trp-supplemented 5%TeaAq containing NEEL19 showed significantly high IAA (18.2 ± 3.49 µg ml^−1^, *P* < 0.0001) as compared to Trp-supplemented cell-free 5%TeaAq (not detectable). Data indicated a prerequisite of cells and Trp for IAA production in liquid cultures. 5%TeaAq^W^ promoted bacterial IAA biosynthesis, whereas 5%OcAq^W^ failed to elicit such a response.

We tested bacterial Trp-dependent IAA production by lowering the doses of OcAq and TeaAq to ≤ 1% (v/v). IAA decreased (4.80 ± 1.45–0.87 ± 0.81 µg ml^−1^) with increasing doses of OcAq (0.25–0.75%, v/v) (Fig. 6c). Significant suppression occurred at 0.75% (v/v) (0.87 ± 0.81 µg ml^−1^, *P* < 0.05) and 1% (v/v) OcAq (not detectable, *P* < 0.01) as compared to OcAq-lacking control (7.92 ± 2.10 µg ml^−1^). In contrast, IAA production increased slightly (6.71 ± 0.76–8.49 ± 1.19 µg ml^−1^) when treated with 0.25–1% (v/v) TeaAq as compared to TeaAq-lacking control (6.63 ± 0.86 µg ml^−1^) (Fig. 6d). These data further substantiated respective suppressive and promotive attributes of OcAq and TeaAq on bacterial Trp-dependent IAA biosynthesis in liquid cultures.

## Discussion

*Pseudomonas* sp. NEEL19 isolated from tea phylloplane shared a high 16S rRNA gene sequence similarity and phylogenetic association with Pseudomonads of plant^36^ and clinical^35,37^ origins. Therefore, experiments were performed on NEEL19 to identify possible plant beneficial features and drug resistance. The co-occurrence of plant growth-promotive traits and antibiotic resistance detected in NEEL19 was in line with our earlier report on a plant-associated bacterium *Burkholderia* sp. LS-044^41^. The ability to utilize diverse compounds as sole carbon and energy sources suggested a remarkable nutritional versatility of NEEL19. Genome sequencing may shed more light on the poorly resolved taxonomic status and genetic make-up of NEEL19.

NEEL19 also exhibited phylogenetic proximity to solvent tolerant *Pseudomonas* strains such as S12^27,38^, BIRD-1^25^, KT2440^39^, VLB120^40^ and IH-2000^28^. Distinguishing growth trends of NEEL19 recorded at 0.5–5% (v/v) ethanol and 1-octanol treatments suggested distinct impacts of these two alcohols on cell growth. Cells were short with a rough surface when treated with 1-octanol, whereas elongated with a smooth surface when exposed to ethanol, in liquid cultures. Ethanol-driven increment in cell size indicated solvent tolerance in NEEL19 since enhancement in cell size was reported to be an adaptive feature of solvent-tolerant strains^26^. In contrast, reduction in cell size presumably provides an increased surface area-to-volume ratio for the transport of long C-chain (≥ 3 C) alcohols.

The tea leaves have been studied extensively for their VOC using various extraction and analytical techniques^1–9,42^. Hydrodistillation in the Clevenger apparatus facilitated the entrapment of tea VOC in aqueous form. While FT-IR provided a preliminary indication of functional groups, GC–MS facilitated specific identification of 1-octanol in TeaAq. Detection of 1-octanol was in line with earlier reports on tea leaves^2–5,9^.

Solvent-tolerant strains have been studied earlier by directly incorporating target solvents into liquid cultures^25–29,38–40^. In contrast, we carried out a detailed investigation on the impact of vapors of volatile compounds on a solvent-tolerant strain besides performing the solvent-emended liquid cultures. AB dye reduction assay, which involves a redox metabolic indicator resazurin^43^, was employed to probe bacterial cell viability, whereas phenol red assay was used to monitor media pH. 1-octanol emitting from TeaAq was identified as one of the responsible molecules modulating bacterial replication and metabolism (including IAA formation) through cell culture and colorimetric assays. Bacteria may produce IAA in Trp-dependent or independent manner^32,34^. Trp boosted the IAA formation in NEEL19 while concurrently suppressing the cell division under OcV and TeaV treatments. Thus, NEEL19 most likely to invest volatile 1-octanol emitting from tea leaves on IAA production than on cell replication in the presence of Trp.

Alcohols promote the biofilm formation in *Pseudomonas* presumably by modifying the cell surface^29^. OcV and TeaV exposures had a similar impact on bacterial cell size, surface features and putative intercellular pili/nanowire formation, indicating a definite role played by 1-octanol in the transformation. Furthermore, OcV was found to promote swimming motility, which influences bacterial colonization and biofilm formation. Bacterial pili act as nanowires promoting cell–cell aggregation and electroactive biofilm formation in *Geobacter*^44^. However, pili of *P. aeruginosa* reported to lack conductance^45^, possibly reflecting their main involvement in motility and establishment of biofilm than electron transport. IAA was reported to induce filamentation in *Saccharomyces cerevisiae*^46^ and promote biofilm formation in *Escherichia coli*^47^. Therefore, the impact of OcV-driven IAA formation on the development of pili/nanowire and biofilm in NEEL19 warrants further investigation.

The impact of the vapors of 1-octanol on NEEL19 was assessed in detail to further ascertain its bioactivity. Cell count, viability, media alkalization and IAA formation were increased in NEEL19 when exposed to OcV in a dose-dependent manner. Thus, for a given amount of volatile 1-octanol emission, solvent-tolerant *Pseudomonas* would produce more biomass and IAA than solvent-sensitive microbiota, possibly manipulating the tea plant physiology and secondary metabolism.

**Figure 7:**
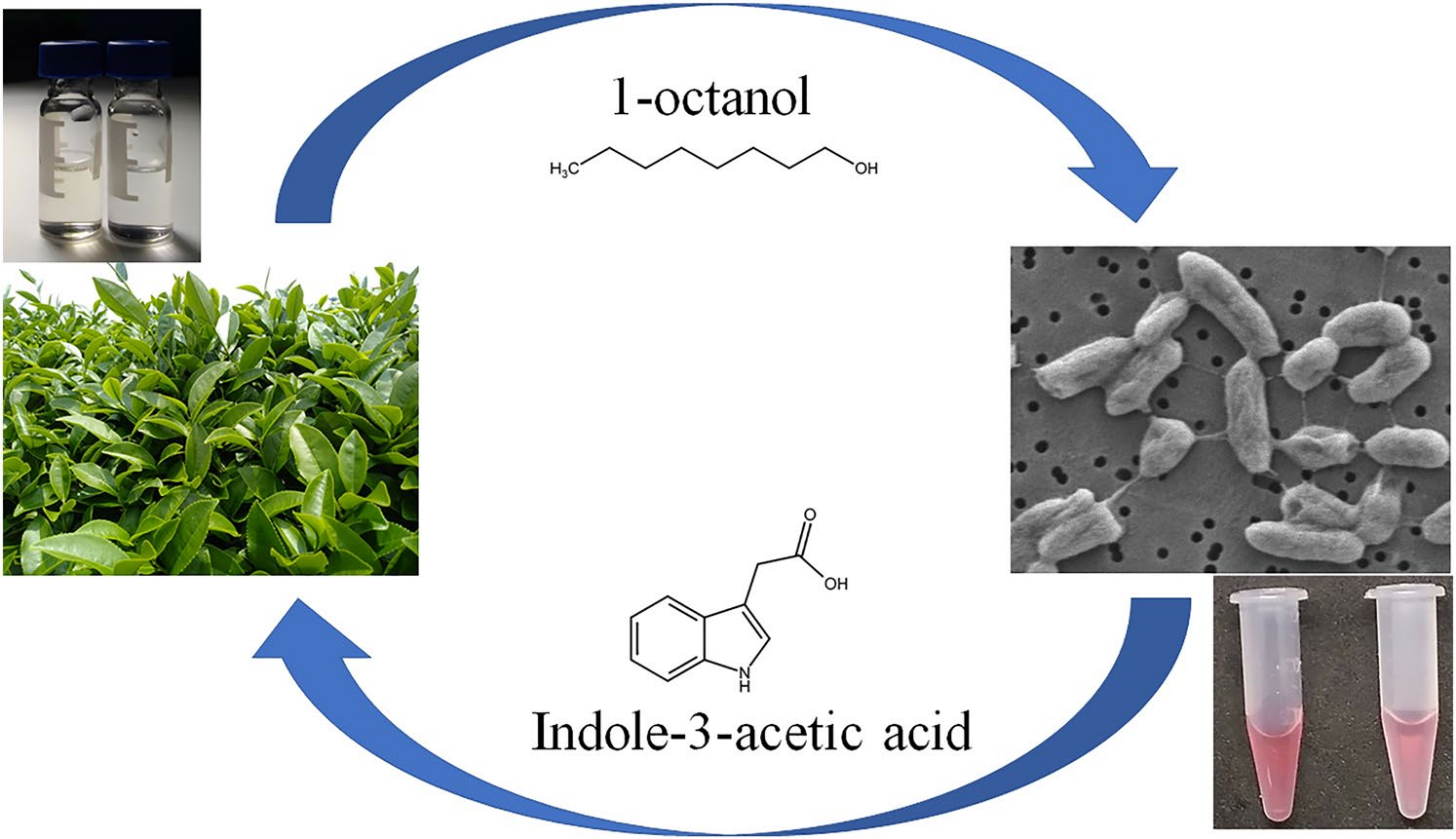
Schematic representation of alcohol-phytohormone exchange occurring between tea phylloplane and its bacterial inhabitant discovered in this study. Images of tea plantation, scanning electron microscopy of NEEL19 and sample vials were from this study.

OcAq failed to trigger bacterial Trp-dependent IAA production, whereas TeaAq boosted the formation of IAA. Results indicated the prerequisite of 1-octanol in vapor form for IAA production. On the other hand, multiple phytochemicals available in TeaAq most likely contributed to IAA formation in liquid cultures. The study highlighted the significance of the volatile micro-niche of tea phylloplane, where alcohol-phytohormone exchange occurs between the host and its inhabitant (Fig. 7).

## Materials and methods

### Reagents and chemicals

High purity (> 99.0%) methanol, ethanol, 1-propanol, 1-butanol and 1-octanol were obtained from Fisher Scientific (Leicestershire, UK). IAA and phenol red were purchased from Sigma Aldrich. AB dye was obtained from Invitrogen.

### Extraction of tea leaf aqueous extract

Fresh and healthy tea leaves were collected from a tea plantation of Nantou County, Yuchi township, Taiwan (23°52′47"N, 120°54′46"E) on 19th November 2018. Leaves (100 g) were introduced into 500 ml deionized water and subjected to conventional hydrodistillation in a Clevenger apparatus (Figure S5) for 2 h according to European Pharmacopoeia method^48^. Aqueous tea extract (TeaAq, 15 ml) was isolated, aliquoted and preserved at − 20 °C.

### FT-IR and GC–MS analysis

TeaAq (250 µl) was mixed with KBr (120 mg) for the preparation of KBr pellets. Samples were analyzed by FT-IR in the standard wavenumber range of 400–4000 cm^−1^ in a PerkinElmer RX1 infrared spectrophotometer. GC–MS analysis was carried out according to earlier descriptions^2^.

### Isolation and identification of *Pseudomonas* sp. NEEL19

Methylotrophic agar was prepared according to the recipe of DSMZ 125 (Deutsche Sammlung von Mikroorganismen und Zellkulturen, Germany) having the following chemical composition (L^−1^ distilled water, pH 6.8): KNO_3_, 1 g; MgSO_4_.7H_2_O, 0.2 g; CaCl_2_.2H_2_O, 0.02 g; Na_2_HPO_4_, 0.23 g; NaH_2_PO_4_, 0.07 g; FeSO_4_.7H_2_O, 1 mg; CuSO_4_.5H_2_O, 5 μg; H_3_BO_3_, 10 μg; MnSO_4_.5H_2_O, 10 μg; ZnSO_4_.7H_2_O, 70 μg, MoO_3_, 10 μg, methanol, 5 ml and agar, 12 g. Adaxial surface of a fresh and healthy tea leaf was briefly impregnated on methylotrophic agar. Inoculated plates were incubated under darkness for 48 h at 30 °C, colonies were picked up and purified. 16S rRNA gene-sequencing and phylogeny were done as described elsewhere^49^. Sequence similarity values were computed using EzBioCloud^50^.

### Plant growth-promotive traits and biochemical features

N_2_ fixation and IAA production were determined by acetylene reduction assay^51,52^ and colorimetry^53^, respectively. Siderophore production was tested on CAS agar^54^. DNase activity was assessed using DNase test agar (Himedia). Catalase and oxidase activities, and hydrolysis of starch (0.2%, w/v) were determined as described elsewhere^55^. Biochemical and enzymatic analyses were performed using API 20 NE, API 20 E and API ZYM (bioMérieux) strips. Antibiotic resistance was tested using ATB staph strip (bioMérieux). Carbon source utilization was determined using GN2 MicroPlate (Biolog). Kit-based tests were done following the manufacturers’ protocol.

### Growth assay for NEEL19 under direct contact of alcohols in liquid cultures

Liquid basal medium (DSMZ 125*) contained the chemical composition of DSMZ 125, except for methanol and agar. Methanol, ethanol, 1-propanol, 1-butanol and 1-octanol at specified doses (0, 0.5, 1, 3 and 5%; v/v) were introduced into DSMZ 125*. Cells were cultured in 4 ml media taken in 20-ml glass test tubes in triplicates (30 °C, 150 rpm, 72 h). OD_600_ was measured using Ultrospec 10 cell density meter (Amersham Biosciences).

### Culture assay for NEEL19 under the supply of carbon sources in vapor form

Colonies were picked up from a 16 h-old nutrient agar (NA; Himedia) plate culture using sterile cotton swabs and washed twice (12,000 × g, 30 °C, 10 min.) in sterile DSMZ 125* without and with 0.1% (w/v) Trp supplement (BM and BM^W^, respectively). Two 500-µl microfuges containing 200 µl culture suspension (BM or BM^W^; 1.0 × 10^6^ ± 3.0 × 10^5^ CFU ml^−1^) were placed at the cell-compartment of PPD (Figure S6). A 500-µl microfuge containing 100 µl of ethanol (EtV), 1-octanol (OcV) or filter-sterilized TeaAq as carbon sources (TeaV) was placed at the volatile carbon-compartment. Inoculated BM^W^ and BM without volatile carbon source supply were served as controls. Dose-dependent analyses for 1-octanol were performed similarly by increasing the amounts of 1-octanol (0, 5, 10, 25, 50, 75, 100, 150 or 200 µl) placed in the volatile carbon-compartment. PPD was tightly sealed with insulation tape and incubated under darkness at 30 °C for 30 h. All culture experiments were performed in quadruplicates.

### Bacterial growth, viability, media acidity/alkalinity and IAA production under the supply of carbon sources in vapor form

Culture microfuges exposed to vapors were retrieved from PPD after 30 h of incubation. Cell suspensions were transferred to a 96-well microplate for OD_600_ measurements in a microplate reader (Biochrom Asys UVM 340). Cells grown on NA (Himedia) were counted (CFU ml^−1^). AB dye reduction (%) was estimated according to the manufacturer’s protocol by introducing 10% (v/v) AB to culture suspension in a microplate, followed by reading the plates at 570 and 600 nm. Media acidity/alkalinity and IAA production were determined in cell-free culture supernatants using microplates. Acidity and alkalinity were probed by adding 10% (v/v) phenol red to the supernatant, and reading the plates at 415 and 560 nm, respectively. IAA production was assessed colorimetrically^53^ with the following modifications: One-fold supernatant was mixed with four-fold Salkowski reagent in a 96-well microplate, incubated for 15 min at room temperature and read at 530 nm. IAA was quantified using a standard curve plotted for the IAA standard.

### Motility assay

Preliminary experiments were carried on full-strength NB (Himedia) supplemented with 0.3, 0.5 and 1.0% (w/v) agar for swimming, swarming and twitching motilities^56,57^, respectively. Subsequently, motility was assessed on PPD pre-casted with 0.3% (w/v) agar-supplemented DSMZ 125* at the cell-compartment. 1-octanol (75 µl) was placed in the volatile carbon-compartment and plates were inoculated and sealed tightly with an insulation tape. Plates were incubated for 72 h at 30 °C and colony diameters were measured. Inoculated 1-octanol-free plates were used as control.

### IAA production in NEEL19 under the direct supply of volatile compounds in liquid cultures

Cell suspensions (1.0 × 10^8^ CFU ml^−1^) were prepared in full-strength nutrient broth (Himedia) without and with Trp (NB and NB^W^, respectively), 5% (v/v)1-octanol in BM and BM^W^ (5%OcAq and 5%OcAq^W^, respectively), 5% (v/v) TeaAq in BM and BM^W^ (5%TeaAq and 5%TeaAq^W^, respectively), 0, 0.25, 0.5, 0.75 and 1% (v/v) 1-octanol in BM^W^ and 0, 0.25, 0.5, 0.75 and 1% (v/v) TeaAq in BM^W^. Two 500 µl microfuge tubes containing 200 µl of the above-mentioned culture suspension were placed in the cell-compartment, whereas the volatile carbon-compartment was left empty. Plates were incubated under darkness at 30 °C for 30 h. IAA was estimated as described above from quadruplicates.

### Scanning electron microscopy

Scanning electron microscopy was performed for 48 h-old cells according to our earlier descriptions^41^.

### Statistical analysis and software

Statistical significance (*P < 0.1, **P < 0.05, ***P < 0.01, ****P < 0.0001) was determined by one-way analysis of variance (ANOVA) with Tukey’s multiple comparisons test using GraphPad Prism (unless specified otherwise). Figures were generated through GraphPad Prism version 6.

Chemical structures and a schematic diagram of the Clevenger apparatus were drawn using Marvin (https://chemaxon.com/products/marvin) and EdrawMax version 9.4 (https://www.edrawsoft.com), respectively.

## Supplementary Information

Supplementary Information.

## Abbreviations

AB: AlamarBlue
BM and BM^W^: NEEL19-inoculated DSMZ 125* without and with 0.1% (w/v) Trp supplement, respectively
BM + EtV, BM + OcV and BM + TeaV: BM exposed to vapors of ethanol, 1-octanol and TeaAq, respectively
BM^W^ + EtV, BM^W^ + OcV and BM^W^ + TeaV: BM^W^ exposed to vapors of ethanol, 1-octanol and TeaAq, respectively
CFU: Colony-forming units
DSMZ 125: Methylotrophic agar prepared according to the recipe of Deutsche Sammlung von Mikroorganismen und Zellkulturen, Germany (DSMZ)
DSMZ 125*: Liquid basal medium (methanol- and agar-free DSMZ 125)
EtV: Ethanol vapor
FT-IR: Fourier transform infrared spectrometry
GC–MS: Gas chromatography-mass spectrometry
IAA: Indole-3-acetic acid
NA: Nutrient agar (Himedia)
NB and NB^W^: Nutrient broth (Himedia) without and with 0.1% (w/v) Trp supplement
OcV: 1-octanol vapor
OD: Optical cell density
PPD: Partition petri dish
TeaAq: Aqueous tea extract
TeaV: TeaAq vapor
Trp: L-tryptophan
VOC: Volatile organic compounds
5%OcAq and 5%OcAq^W^: 5% (v/v) 1-octanol-supplemented BM and BM^W^, respectively
5%TeaAq and 5%TeaAq^W^: 5% (v/v) TeaAq-supplemented BM and BM^W^, respectively
OcAq^W^ and TeaAq^W^: BM^W^ containing specified doses (%, v/v) of OcAq and TeaAq, respectively
