# Supplementary material for: Volatile 1-octanol of tea (Camellia sinensis L.) fuels cell division and indole-3-acetic acid production in phylloplane isolate Pseudomonas sp. NEEL19
Source: Sci Rep. 2021 Feb 2;11:2788. doi: 10.1038/s41598-021-82442-7 (PMC7854675; doi:10.1038/s41598-021-82442-7)
Supplement: Supplementary file 1 — Supplementary Information. [file 41598_2021_82442_MOESM1_ESM.docx]

**Supplementary Material**

**Volatile 1-octanol of tea (*Camellia sinensis* L.) fuels cell division and indole-3-acetic acid production in phylloplane isolate *Pseudomonas* sp. NEEL19**

Poovarasan Neelakandan^1^, Chiu-Chung Young^1,2,^*, Asif Hameed^1,3^, Yu-Ning Wang^1^, Kui-Nuo Chen^1^ & Fo-Ting Shen ^1,2,^*

^1^*Department of Soil & Environmental Sciences, College of Agriculture and Natural Resources, National Chung Hsing University, Taichung 40227, Taiwan, R.O.C.*

^2^*Innovation and Development Center of Sustainable Agriculture (IDCSA), National Chung Hsing University, Taichung 40227, Taiwan, R.O.C.*

^3^*Yenepoya Research Centre, Yenepoya Deemed to be University, Mangalore 575018, India.*

***Corresponding authors:**

Prof. Fo-Ting Shen

Tel.: +886-4-22840373#4306; Fax: 886-4-22854250

Email: [ftshen@dragon.nchu.edu.tw](mailto:ftshen@dragon.nchu.edu.tw)

Prof. Chiu-Chung Young

Tel.: 886-4-22861495; Fax: 886-4-22861495

E-mail: [ccyoung@mail.nchu.edu.tw](mailto:ccyoung@mail.nchu.edu.tw)

**Biochemical and enzymatic assay data of *Pseudomonas* sp. NEEL19**

In API 20 NE, negative for indole production, fermentation of glucose, urease, hydrolysis of esculin, gelatin and *p*-nitrophenyl-*β-*D-galactopyranoside; assimilation of D-mannitol, *N*-acetyl-glucosamine, D-maltose, adipic acid and phenylacetic acid. In API 20 E, negative for hydrolysis of *o*-nitrophenyl-*β-*D-galactopyranoside, lysine decarboxylase, ornithine decarboxylase, H_2_S production, urease, tryptophan deaminase, indole production, acetoin production, gelatinase, fermentation/oxidation of D-mannitol, inositol, D-sorbitol, L-rhamnose, D-sucrose and amygdalin. In API ZYM, negative for valine arylamidase, cystine arylamidase, trypsin, *α*-chymotrypsin, *α*-galactosidase, *β*-galactosidase, *β*-glucuronidase, *α*-glucosidase, *N*-acetyl-*β*-glucosaminidase, *α*-mannosidase and *α*-fucosidase. In Biolog GN2 MicroPlate, positive for the oxidation of dextrin, glycogen, Tween 40, Tween 80, *N*-acetyl-D-glucosamine, adonitol, L-arabinose, D-arabitol, D-fructose, L-fucose, D-galactose, gentiobiose, *α*-D-glucose, m-inositol, *α*-D-lactose, lactulose, maltose, D-mannitol, D-mannose, D-melibiose, D-psicose, D-sorbitol, sucrose, turanose, xylitol, pyruvic acid methyl ester, succinic acid mono-methyl ester, acetic acid, *cis*-aconitic acid, citric acid, formic acid, D-galactonic acid lactone, D-galacturonic acid, D-gluconic acid, D-glucosaminic acid, D-glucuronic acid, *β*-hydroxybutyric acid, *γ*-hydroxybutyric acid, , *α*-keto glutaric acid, D,L-lactic acid, malonic acid, propionic acid, quinic acid, D-saccharic acid, sebacic acid, succinic acid, bromosuccinic acid, succinamic acid, glucuronamide, L-alaninamide, D-alanine, L-alanine, L-alanyl glycine, L-asparagine, L-aspartic acid, L-glutamic acid, glycyl-L-aspartic acid, glycyl-L-glutamic acid, L-histidine, hydroxy-L-proline, L-ornithine, L-proline, L-pyroglutamic acid, L-serine, *γ*-amino butyric acid, urocanic acid, inosine, putrescine, 2-aminoethanol, 2,3-butanediol, glycerol, D,L-*α*-glycerol phosphate, *α*-D-glucose-1-phosphate and D-glucose-6-phosphate; negative for the oxidation of *α*-cyclodextrin, *N*-acetyl-D-galactosamine, D-cellobiose, i-erythritol, *β*-methyl-D-glucoside, D-raffinose, L-rhamnose, D-trehalose, *α*-hydroxybutyric acid, *p*-hydroxy phenylacetic acid, itaconic acid, *α*-keto butyric acid, *α*-keto valeric acid, L-leucine, L-phenylalanine, D-serine, L-threonine, D,L-carnitine, uridine, thymidine and phenyethylamine,

**Table S1.** Closest (≥99.0%, 16S rRNA gene) hits obtained for *Pseudomonas* sp. NEEL19 at EzBiocloud server

| **Rank** | **Name** | **Strain** | **Accession** | **Similarity* (%)** |
| --- | --- | --- | --- | --- |
| 1 | *Pseudomonas juntendi* | BML3^T^ | MK680061 | 100.0 |
| 2 | AE015451_s | KT2440 | AE015451 | 99.9 |
| 3 | *Pseudomonas hunanensis* | LV^T^ | JX545210 | 99.9 |
| 4 | *Pseudomonas shirazica* | VM14^T^ | OLKI01000048 | 99.8 |
| 5 | *Pseudomonas asiatica* | RYU5^T^ | MH517510 | 99.8 |
| 6 | NEIG_s | R17(2017) | NEIG01000032 | 99.8 |
| 7 | *Pseudomonas alloputida* | Kh7^T^ | LT718459 | 99.8 |
| 8 | *Pseudomonas taiwanensis* | BCRC 17751^T^ | EU103629 | 99.8 |
| 9 | CP026115_s | W5 | CP026115 | 99.7 |
| 10 | *Pseudomonas monteilii* | NBRC 103158^T^ | BBIS01000088 | 99.7 |
| 11 | *Pseudomonas plecoglossicida* | NBRC 103162^T^ | BBIV01000080 | 99.7 |
| 12 | AKJC_s | GM84 | AKJC01000277 | 99.6 |
| 13 | QJRL_s | LB-090624 | QJRL01000068 | 99.5 |
| 14 | *Pseudomonas parafulva* | NBRC 16636^T^ | BBIU01000051 | 99.4 |
| 15 | *Pseudomonas mosselii* | CIP 105259^T^ | AF072688 | 99.4 |
| 16 | *Pseudomonas entomophila* | L48^T^ | CT573326 | 99.4 |
| 17 | CP000926_s | GB-1 | CP000926 | 99.4 |
| 18 | LMLL_s | Leaf58 | LMLL01000001 | 99.4 |
| 19 | CP024085_s | E41 | CP024085 | 99.4 |
| 20 | CP025262_s | 02C 26 | CP025262 | 99.3 |
| 21 | *Pseudomonas fulva* | NBRC 16637^T^ | BBIQ01000036 | 99.2 |
| 22 | *Pseudomonas capeferrum* | WCS358^T^ | JMIT01000002 | 99.2 |
| 23 | *Pseudomonas sichuanensis* | WCHPs060039^T^ | QKVM01000121 | 99.2 |
| 24 | LBME_s | ATH-43 | LBME01000002 | 99.2 |
| 25 | CP005976_s | H8234 | NC_021491 | 99.2 |
| 26 | *Pseudomonas putida* | NBRC 14164^T^ | AP013070 | 99.1 |
| 27 | CP013997_s | USDA-ARS-USMARC-56711 | CP013997 | 99.1 |
| 28 | *Pseudomonas guariconensis* | LMG 27394^T^ | FMYX01000029 | 99.0 |
| 29 | *Pseudomonas reidholzensis* | CCOS 865^T^ | LT009707 | 99.0 |
| 30 | BATF_s | ICMP 17674 | BATF01000024 | 99.0 |
| 31 | CP027706_s | CMR12a | CP027706 | 99.0 |
| 32 | CP016634_s | IEC33019 | CP016634 | 99.0 |
|  | *Pair-wise |  |  |  |

**Table S2.** Antibiotic (ATB STAPH 5) assay results obtained for *Pseudomonas* sp. NEEL19

| **Antibiotic** | **Abbreviation** | **Concentration (mg L^‒1^)** | **NEEL19** |
| --- | --- | --- | --- |
| Penicillin | PEN | 0.12 | R |
| Cotrimoxazol | TSU | 2/38 | R |
| Gentamicin | GEN | 4‒8 | I |
| Erythromycin | ERY | 0.5‒4 | R |
| Clindamycin | CLI | 0.5‒2 | R |
| Tetracycline | TET | 4‒8 | R |
| Minocycline | MIN | 4‒8 | R |
| Vancomycin | VAN | 4‒16 | R |
| Teicoplanin | TEC | 8‒16 | R |
| Rifampicin | RFA | 1‒2 | R |
| Norfloxacin | NOR | 4‒8 | I |
| Levofloxacin | LVX | 2‒4 | S |
| Fusidic acid | FUC | 2‒16 | R |
| Nitrofurantoin | FUR | 32‒64 | R |
| Quinupristin-Dalfopristin | QDA | 1‒2 | R |
| Coag(-) Oxacillin | OXAE | 0.25 | R |
| Oxacillin | OXA | 2 | R |

R, Resistant; S, Sensitive; I, Intermediate response

**Table S3.** Prediction of some of the key compounds present in TeaAq through GC-MS/FID analysis

| Compound | Database |
| --- | --- |
| 3-Hexen-1-ol, (Z)- | NIST98.L |
| 4-Hexen-1-ol, (Z)- | NIST98.L |
| Formic acid, octyl ester | NIST98.L |
| Heptacosane | NIST98.L |
| **1-Octanol** | **NIST98.L** |
| Cis-linalool oxide | WILEY275.L |
| 2,6-Octadienal, 3,7-dimethyl- | NIST98.L |
| .alpha.-longipinene | WILEY275.L |
| 1,4-Dimethyl-1,2,3,4-tetrahydronaphthalene | WILEY275.L |
| Nonadecane | NIST98.L |
| 2-[(phenylsulfonyl)methyl]skatole | WILEY275.L |
| 4,5-dehydro-isolongifolene | WILEY275.L |
| Tetradecane | NIST98.L |
| Methyl ester of 3,4,5-trichloro-6-(p-toluoyl) picolinic acid | WILEY275.L |

**Supplementary figure captions**

**Figure S1.** Unrooted neighbor-joining phylogenetic tree based on 16S rRNA gene sequences showing the position of *Pseudomonas* sp. NEE19 (1438 bp) and other related strains of genus *Pseudomonas*. Type strains are indicated by superscript ‘T’. Solvent-tolerant strains described earlier are shown in blue-fonts. Strains originated from plant and clinical sources are shown in green- and red-fonts, respectively. Bootstrap values (>70 %) based on 1000 replications are shown at the nodes. The GenBank/EMBL/DDBJ accession number of each sequence is shown in parentheses. Bar, 0.010 substitutions per nucleotide position.

**Figure S2.** Siderophore production and DNase assay results obtained for *Pseudomonas* sp. NEEL19. (**a**) Orange halo showing siderophore production on CAS agar. (**b**) Lack of halo on DNase test agar indicates negative reaction. Plates were incubated at 30 °C for 48 h. The images were from this study.

**Figure S3.** The influence of the direct contact of 0.5% (v/v) alcohols when supplied as sole carbon and energy sources in liquid basal medium (DSMZ 125*) on cell morphology of *Pseudomonas* sp. NEEL19. Scanning electron micrographs of cells grown in (**a**) methanol, (**b**) ethanol, (**c**) 1-propanol, (**d**) 1-butanol and (**e**) 1-octanol taken after 72-h of culture.

**Figure S4.** The influence of the direct contact of 0.5% (v/v) alcohols when supplied as sole carbon and energy sources in liquid basal medium (DSMZ 125*) on cell size of *Pseudomonas* sp. NEEL19. Variation in (**a**) cell length and (**b**) width are shown. Ethanol 0.5% (v/v)-treated cells were used as controls. Error bar, mean (n=60) ± s.d. **P*<0.1, ****P*<0.01, *****P*<0.0001; ns, non-significant.

**Figure S5.** Schematic diagram of the Clevenger apparatus used for hydrodistillation and isolation of crude aqueous extract of tea leaves (TeaAq). The diagram was drawn using EdrawMax version 9.4 (<https://www.edrawsoft.com>).

**Figure S6.** Placement of volatile carbon sources and cell suspension (BM/BM^W^) in carbon-compartment and cell-compartment, respectively. Culture suspension was pre-stained with 10% phenol red (v/v) for presentation. The image was from this study.

**Figure S7.** Preliminary motility assay results and the influence of vapors of 1-octanol (OcV) on the motility of *Pseudomonas* sp. NEEL19. Motility observed at (**a**) 0.3%, (**b**) 0.5% and (**c**) 1.0% agar-containing full-strength NA (Himedia) after 36 h incubation at 30 °C. Swimming motility in the (**d**) absence (control) and (**e**) presence of 1-octanol vapor (OcV) observed at PPD containing liquid basal medium (DSMZ 125*) supplemented with 0.3% agar after 60 h of incubation. (**f**) Bar diagram showing the promotive influence of OcV on swimming motility. The images were from this study. Error bar, mean (n=4) ± s.d. **P*<0.1.


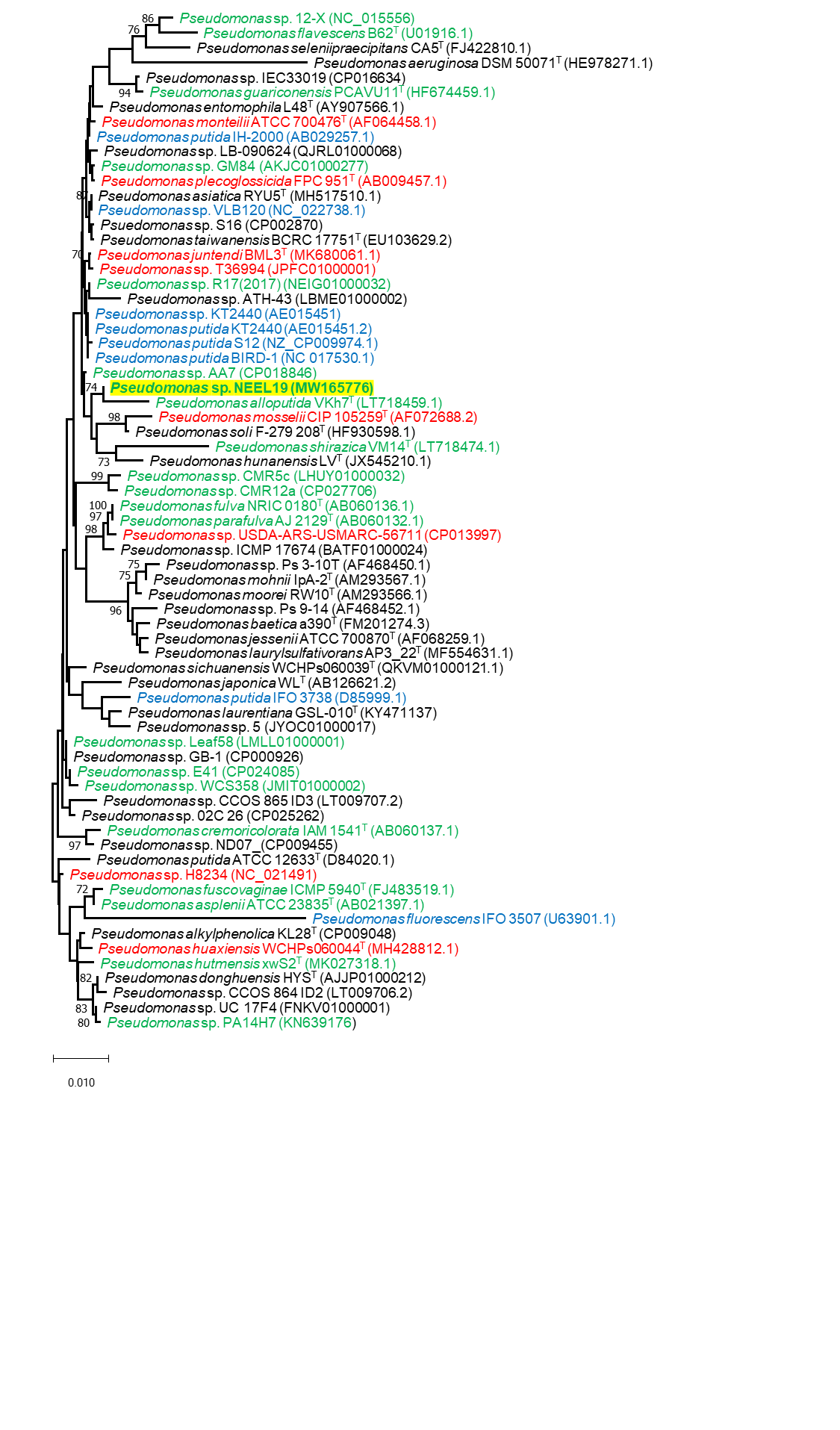


**Figure S1**

(a) (b)


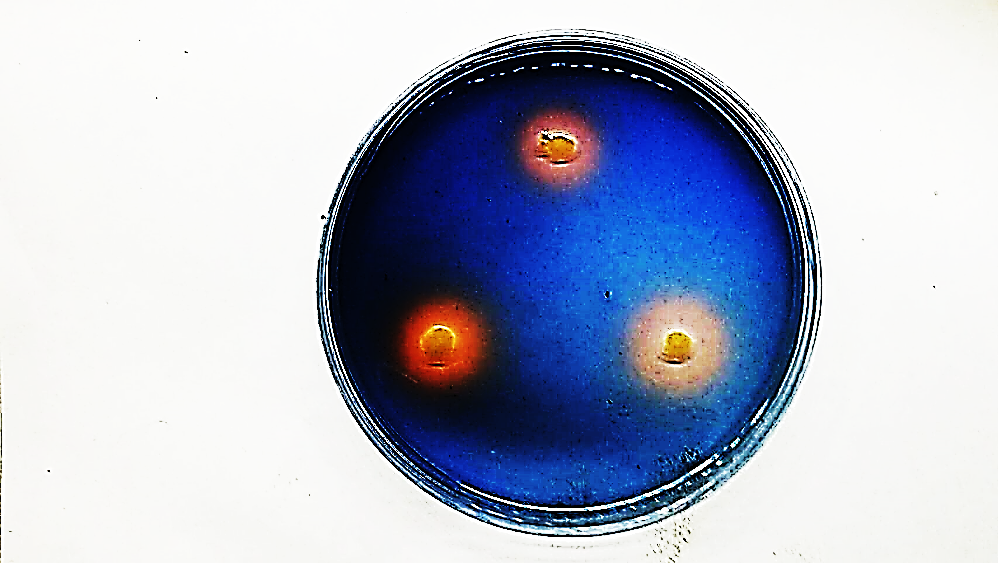

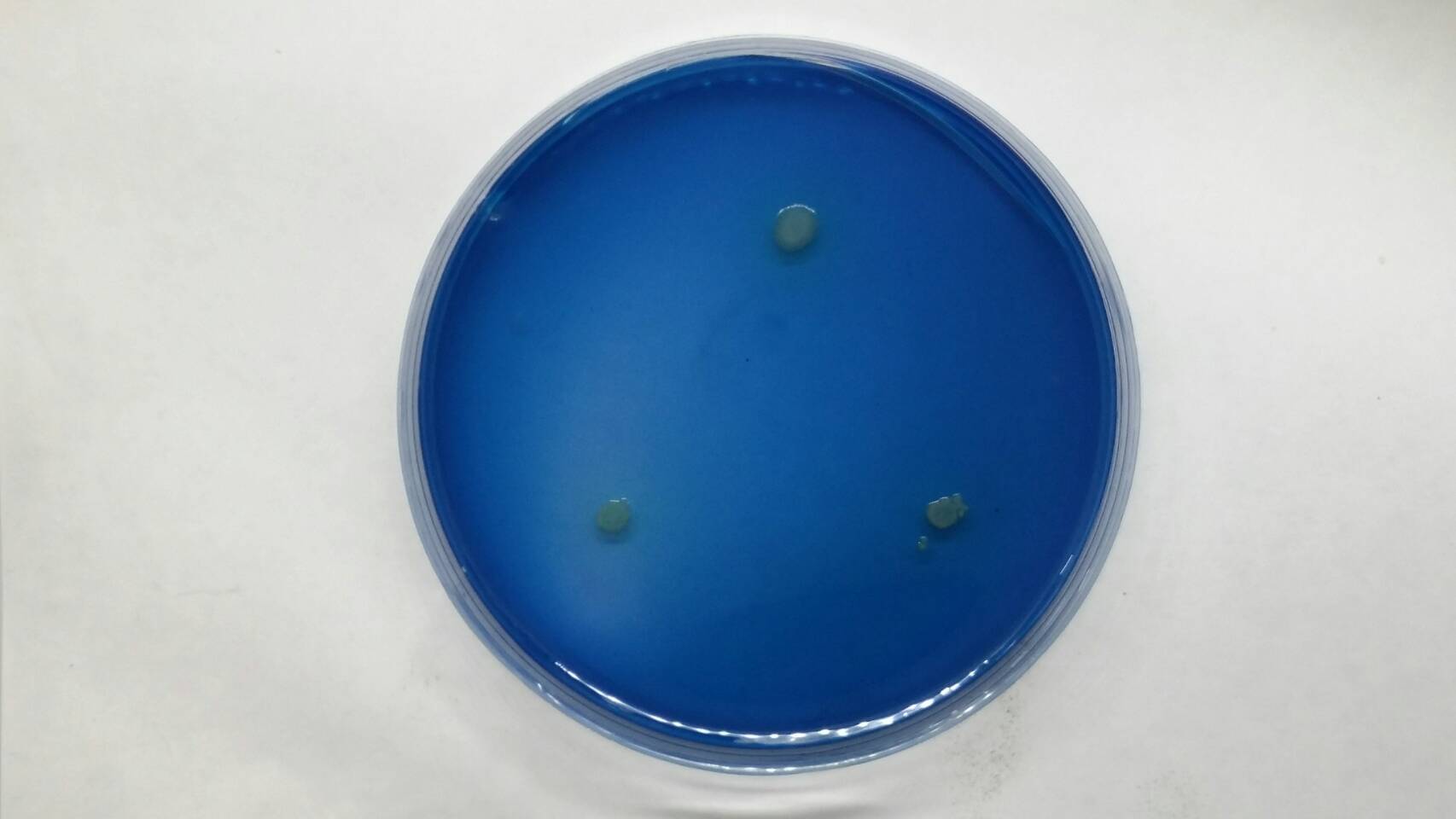


**Figure S2**

(a) (b) (c)


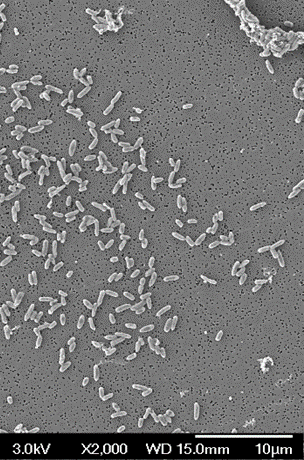

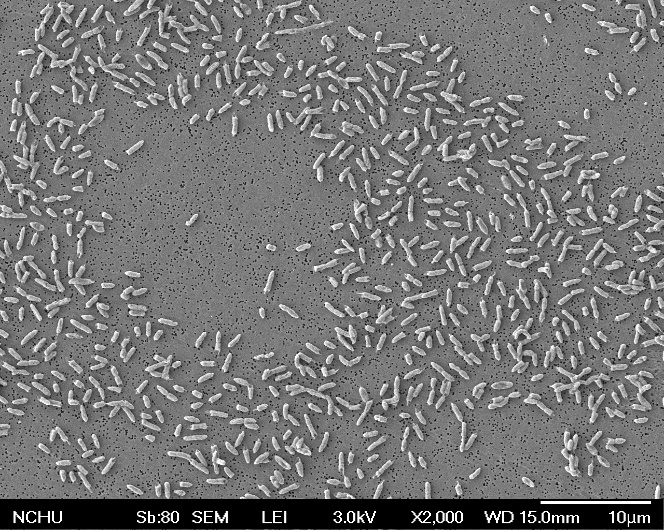

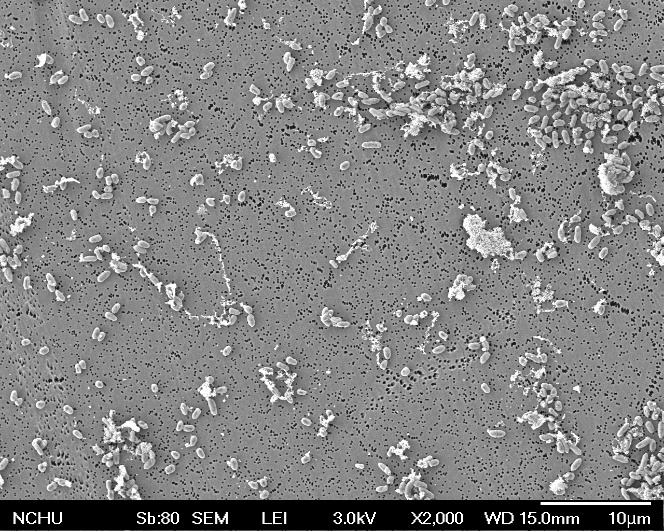


(d) (e)


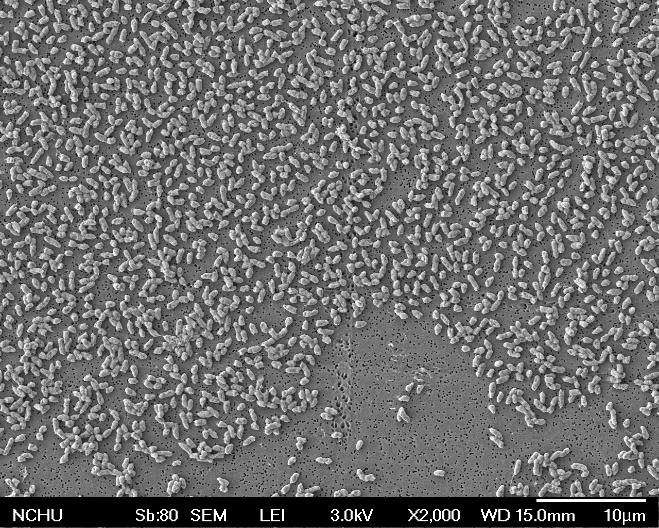

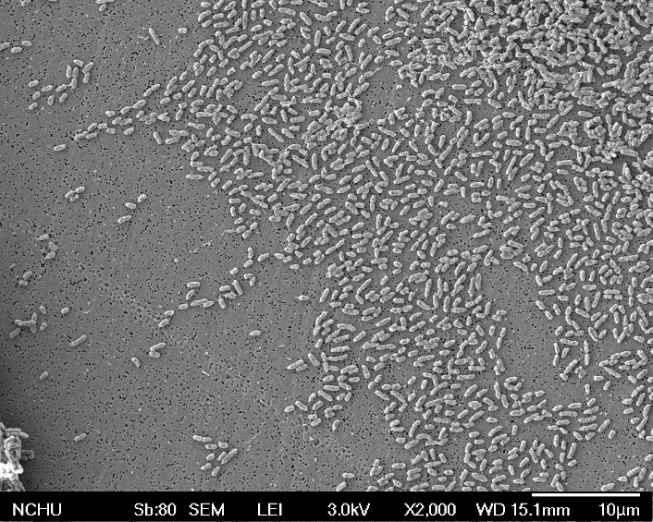


**Figure S3**

(a)

(b)

**Figure S4**


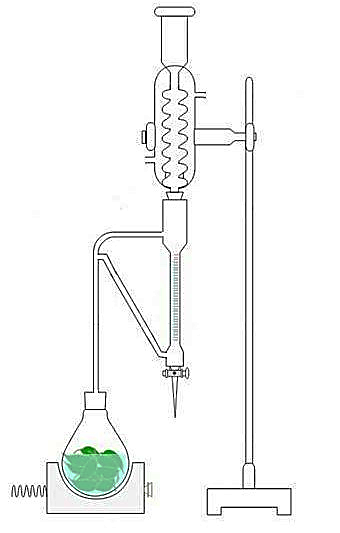


**Figure S5**


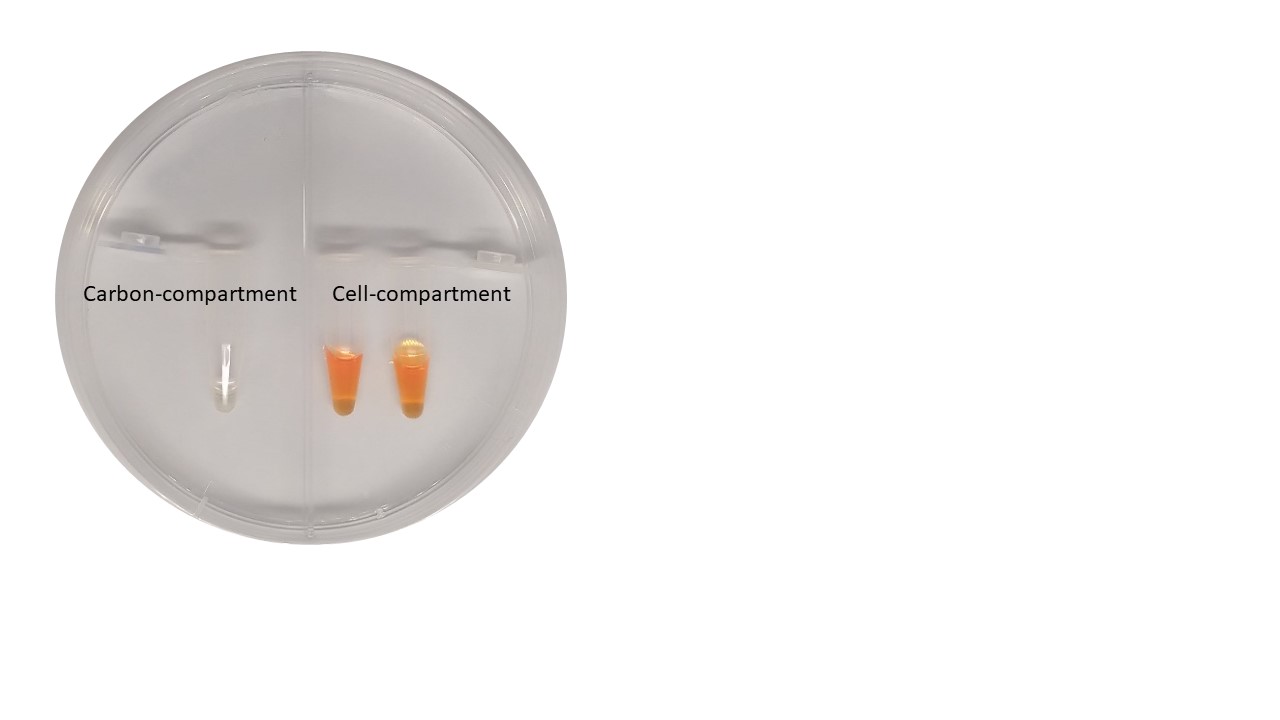


**Figure S6**

1. (b) (c)


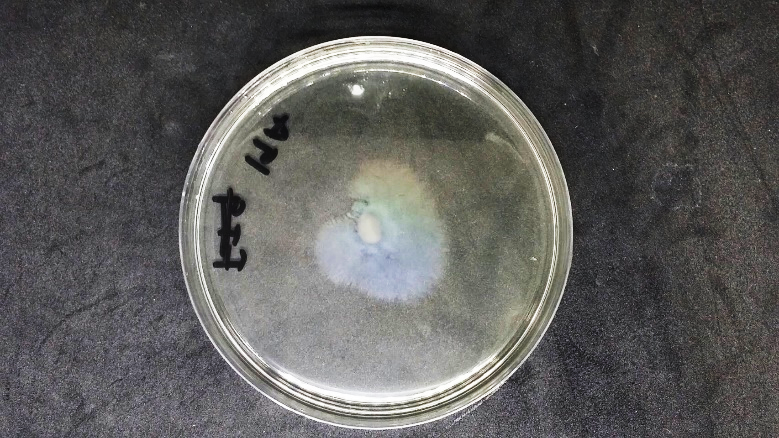

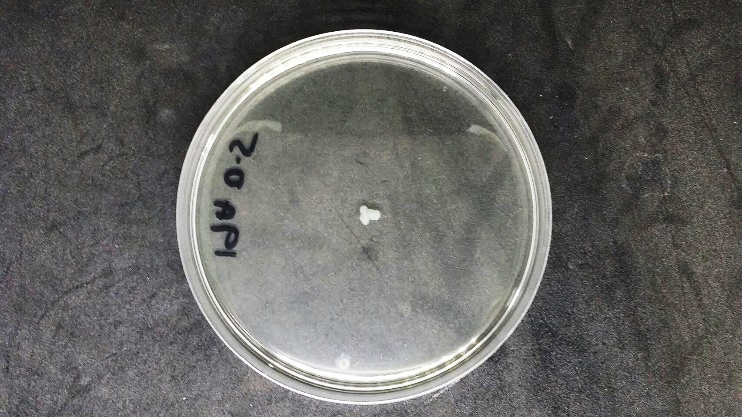

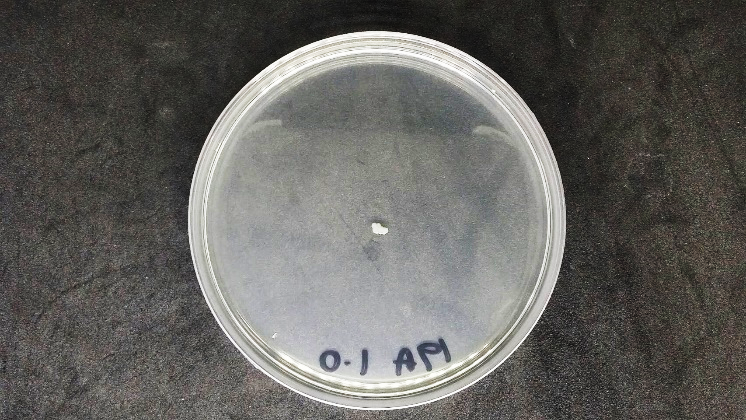


(d) (e)


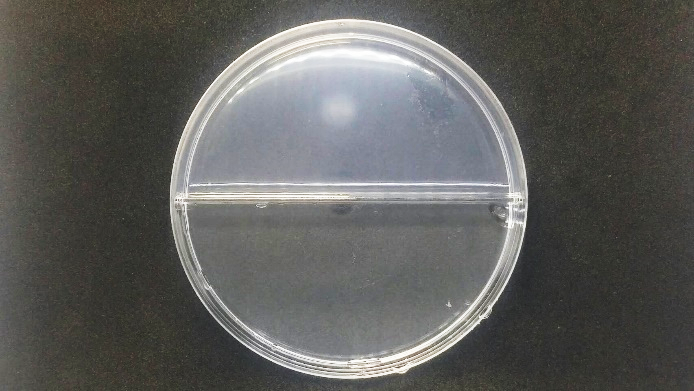

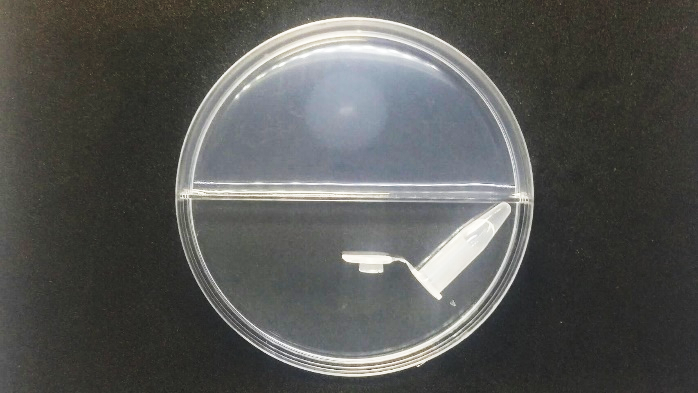


(f)

**Figure S7**
